# Iron accumulation deteriorated bone loss in estrogen-deficient rats

**DOI:** 10.1186/s13018-021-02663-4

**Published:** 2021-08-24

**Authors:** Lu-lin Liu, Gong-wen Liu, Hui Liu, Kai Zhao, You-jia Xu

**Affiliations:** 1 Department of Orthopedics, The First Affiliated Hospital of Gannan Medical University, Ganzhou, 341000 Jiangxi China; 2 Suzhou TCM Hospital Affiliated to Nanjing University of Chinese Medicine, Suzhou, 215009 Jiangsu China; 3 Department of Orthopedics, Ganxian District Traditional Chinese Medicine Hospital of Ganzhou City, Ganzhou, 341100 Jiangxi China; 4 Department of Orthopedics, Second Affiliated Hospital of Soochow University, 1055 Sanxiang Road, Suzhou, 215004 Jiangsu China

**Keywords:** Iron overload, Ovariectomized rat, Osteoporosis, Micro-CT, Iron metabolism, Biomechanical testing

## Abstract

**Background:**

Postmenopausal osteoporosis is characterized by an imbalance of bone resorption exceeding bone formation, resulting in a net loss of bone mass. Whether a menopause-related excess of iron contributes to the development of postmenopausal osteoporosis has remained unresolved due to a lack of an appropriate animal model. This study aimed to explore the effects of iron accumulation in bone mass in estrogen-deficient rats.

**Methods:**

In the present study, ovariectomy (OVX) was performed in female rats and the changes of iron metabolism and some related modulated genes were detected. Ferric ammonium citrate (FAC) was used as a donor of iron for OVX rats. Moreover, micro-CT was performed to assess the bone microarchitecture in sham group, OVX, and FAC groups. Histological detection of iron in liver was assessed by Perl’s staining. The expressions of β-CTX and osteocalcin were assessed by ELISA.

**Results:**

It was found that serum iron decreased after OVX. It was found that the expressions of *Hepcidin* in liver and *Fpn*, *DMT-1* in duodenum significantly decreased at transcriptional level in OVX group than sham group. However, no difference existed in the expression of *DMT-1*. Then, ferric ammonium citrate (FAC) was used as a donor of iron for OVX rats. The FAC group manifested significant iron accumulation by increased serum iron and hepatic iron content. In addition, FAC treatment accelerated bone loss and decreased BMD and biomechanics in OVX rats. Moreover, bone biomarker β-CTX rather than osteocalcin increased significantly in FAC groups than OVX group.

**Conclusions:**

In conclusion, no iron accumulation occurred in OVX rats. Furthermore, iron accumulation could further deteriorate osteopenia through enhanced bone resorption.

## Introduction

As an essential element for human metabolism, iron plays a critical role in many biological processes, such as oxygen transport and enzymatic reactions [1, 2]. Recently, preclinical and clinical studies demonstrated a close relationship between bone metabolism and iron homeostasis. In 2006, Weinberg et al. noted that iron overload was a risk factor for osteoporosis [3]. Some researchers reported that osteoporosis was a common complication in patients with disorders of iron overload [4–6]. In healthy populations, serum ferritin levels were also positively correlated with accelerated bone loss in femur [7].

As a common disease characterized by low bone mineral density (BMD) and a high incidence of bone fractures, osteoporosis occurred in about half of women aged over 50 years [8]. A study indicated that 1 year of menstrual blood for a woman was equivalent to a discharge of 36 mg iron. Several years after menopause, women often experienced iron accumulation as a result of ceased menstruation [9], and the serum level of ferritin increased two- to threefold from before menopause to postmenopause [9]. It is hypothesized that, besides estrogen deficiency, a menopause-related excess of iron could be a risk factor affecting the health of postmenopausal women [10].

Thus, it is practically meaningful to establish an experimental animal model mimicking the postmenopausal status characterized by estrogen deficiency concurrently with iron overload. However, unlike primates, some commonly used experimental rodents such as rats and mice lacked spontaneous decidualization and menstruation [11, 12], and the ovariectomized rats demonstrated decreased estrogen but not iron accumulation [13, 14]. The present study was conducted to verify whether ovariectomy affected iron homeostasis. Furthermore, for the purpose of mimicking the postmenopausal status, ferric ammonium citrate (FAC) was used as a donor of iron for ovariectomized rats, and the effect of iron on bone metabolism was examined.

## Materials and methods

### Experimental design

Sixty-four Sprague-Dawley rats (3 months old, weighing 250 ± 20 g) were used. The protocol was approved by our institutional Ethics Committee for Laboratory Animal Experiments. Utmost care was taken to minimize discomfort, distress, and pain to animals. The rats were randomly divided into groups of sham-operated (sham), ovariectomized (OVX), and OVX-control, and 90 or 180 mg/kg ferric ammonium citrate (FAC) was administered as iron donor (FAC1 and FAC2 respectively). An intraperitoneal injection of normal saline or FAC was dosed at 1-week post-operation, twice weekly for 12 weeks. The rats were weighed weekly and doses adjusted according to weight. At necropsy, intra-cardiac blood was collected under chloral hydrate anesthesia. After centrifugation, sera was harvested and preserved at −80 °C until analysis. Liver was flushed by phosphate buffered saline (PBS) for about 5 min and collected. The right femur and tibia were dissected and stored along with liver in a freezer at −80 °C until examination.

### Serum, liver, and bone mineral content measurement

Serum iron was determined by atomic absorption spectrophotometer. Tibial iron content was measured by inductively coupled plasma-optical emission spectrometry (ICP-OES) as reported previously [15]. Briefly, the weight of each tibia was determined, and the representative samples were placed in tarred porcelain crucibles and dried at 110 °C overnight. After dry weight was determined, they were baked in a Muffle furnace at 550 °C. Ash was dissolved in 3N hydrogen chloride and its total iron content determined by atomic absorption. Mineral content was calculated as the ratio of ash weight to dry weight.

### Determination of Hepcidin, FPN, DMT-1, and DCTYB mRNA expression

Total RNA was extracted from liver and duodenum using Trizol reagent according to the manufacturer’s instructions (Invitrogen, USA). cDNA synthesis was performed with 1 μg RNA using ReverAidTM First Strand cDNA Synthesis Kit (Fermentas K1622, Canada). The oligo-nucleotide primers used were listed in Table 1. Equal amounts of each reverse-transcription product (1 μg) were PCR-amplified using DreamTaqTM Green PCR Master Mix (2X) (Fermentas K1081, Canada) for 30 cycles consisting of 1 min at 95 °C, 30 s at 55 °C, and 1 min at 72 °C. The amplified cDNA was run on 1% agarose gel and visualized under UV light.

**Table 1:** Primers sequences with their corresponding RT-PCR product size

Gene primer	Sequence	Size (bp)
GAPDH	Forward:5′-GACATGCCGCCTGGAGAAAC-3′ Reverse:5′-AGCCCAGGATGCCCTTTAGT-3′	128
Hepcidin	Forward:5′-ACAGAAGGCAAGATGGCACT-3′ Reverse:5′-GAAGTTGGTGTCTCGCTTCC-3′	201
FPN	Forward:5′-TACGGAAACAGCCTCCTCTT-3′ Reverse:5′-GCATTCTTATCCACCCAGTCA-3′	101
DMT-1	Forward:5′-AGCCATCAGAGCCAGTGTGT-3′ Reverse:5′-CCCCAGTGTTTCCCAACTAA-3′	111
DCYTB	Forward:5′-TCTCTTCCAAAGTCAGCCCTAC-3′ Reverse:5′-CTCAGTCAACACAATCGCTCTC-3′	107

### Determination of E2 and bone turnover markers in serum by ELISA

Serum E2 was determined by Quantitative Rat Competitive ELISA (IDS, USA). Plasma osteocalcin (BGP), a marker of bone formation, was measured with a murine osteocalcin EIA kit (Biomedical Technologies, Inc., Stoughton, MA, USA). The serum marker of bone resorption β-CTX was measured with a commercial murine β-CTX ELISA kit (Immunodiagnostic systems Ltd., Boldon, UK). The measurement was performed in 96-well microplates coated with affinity-purified anti-mouse ferritin (Abnova, Taiwan of China). All ELISAs were performed according to the supplier’s instructions.

### Perls’ stain of liver

For visualizing ferric iron deposits, Perls’ staining was performed on liver tissues. After fixing in 10% buffered formalin and embedding in paraffin, Perls’ Prussian blue staining was used for detection.

### Trabecular bone microarchitecture

Densitometric and morphometric micro-CT was performed on trabecular bone from the distal third of femurs. Briefly, bones were positioned with gauze in the sample holder and allowed to reach room temperature. The samples were analyzed on a Skyscan 1176 (Skyscan, Aartselaar, Belgium) using an 18-μm voxel size, 59 KVp, 127 uA, 0.4° rotation three-dimensional reconstruction, and data processing. The trabecular region of interest (ROIs) extended from 50 μm proximally to the end of distal growth plate over 1.7 mm toward diaphysis. The defining process was conducted by a processing system. Trabecular ROIs were drawn freehand on sequential slices to include endosteal envelope, conforming to the endosteal contour on each slice. A global threshold was defined as the lowest mineral density from all bones analyzed, for which volumes were noise-free and trabecular appeared to have the same width in binarized images as in gray-scale image. Bone mineral density (BMD) measurements were performed on cortical and trabecular volumes after segmentation of bone voxels using the global threshold. Only bone slices were included. For other trabecular morphometric measurements, the volumes were binarized using the same threshold as in the BMD measurement.

### Bone mechanical testing

Before mechanical testing, left femur was thawed slowly at room temperature for 2 h and removed from gauze wraps. The samples were tested under three-point bending test using a materials testing machine Zwick-Z010 testing systems (Z010; Zwick GmbH, Ulm, Germany) as previously described [16]. Briefly, each femur was placed in material test machine with two support points separated by a distance of 20 mm. The biomechanical quality of femoral diaphysis was determined at a speed of 2 mm/min. The central loading point was displaced, and load and displacement were recorded until samples became broken. From the load-deformation curve, maximum load, maximum stress, and Young’s modulus were obtained.

### Data analysis

Data management and statistical analysis were conducted with SPSS version 15.0.1 for Windows. The data followed a normal or near normal distribution and were expressed as mean ± standard deviation. One-way analysis of variance was used to evaluate the statistically significant differences among four groups, and *p* value < 0.05 was considered statistically significant.

## Results

### Serum E2 and iron concentration and tibial iron content in sham and OVX groups

**Fig. 1:**
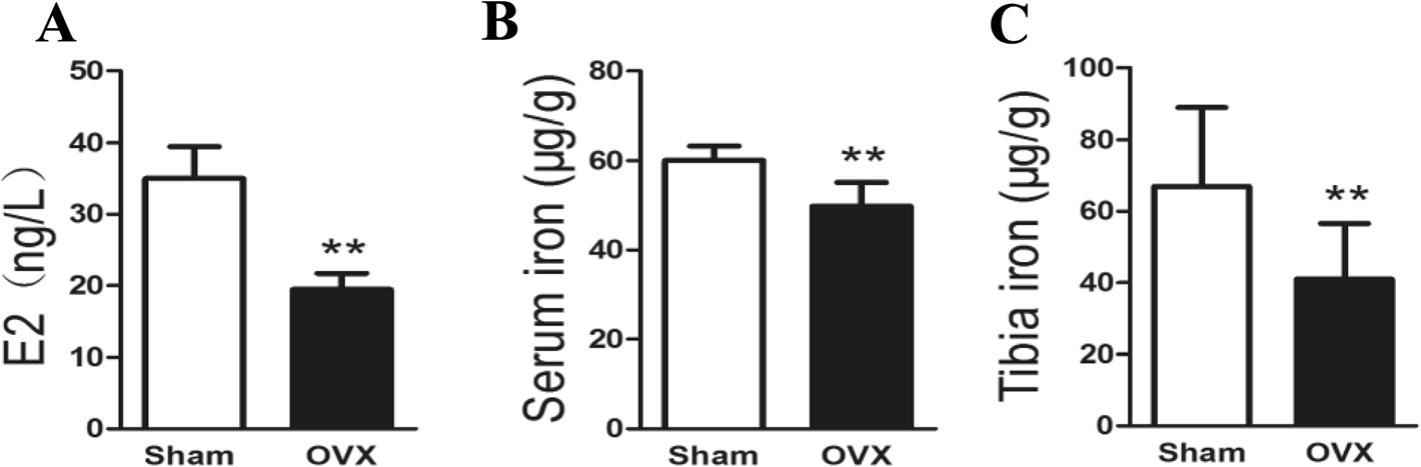
Parameters of serum E2 (**A**), serum iron (**B**), and tibial iron content (**C**) in sham-operated (sham) and ovariectomized (OVX) rats. The bars represented mean ± SD. *p < 0.05, vs. sham group

As compared with sham group, serum estrogen content decreased significantly after ovariectomy. However, with the decrease of estrogen, the serum and tibial iron levels also decreased after ovariectomy (Fig. 1A, B, and C).

### Effects of ovariectomy on the Hepcidin expression in liver and the expressions of Fpn, DMT-1, and DCYTB in duodenum

**Fig. 2:**
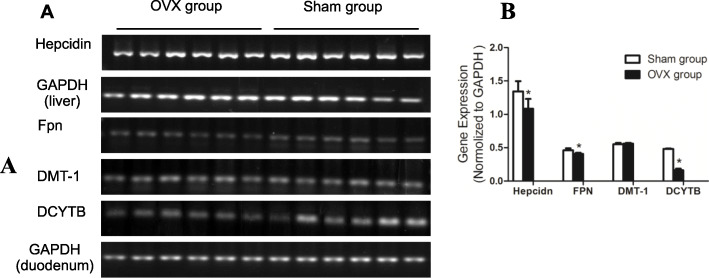
Gene expression of Hepcidin in liver, Fpn, DMT-1, and DCYTB in duodenum of sham and OVX groups. **A** RT-PCR amplification of Hepcidin, GAPDH, Fpn, DMT-1, and DCYTB. **B** The mRNA expressions of Hepcidin, Fpn, DMT-1, and DCYTB. *p < 0.05, versus sham group

As shown above, the serum and tibial iron level decreased after ovariectomy. For the question as to whether the declined estrogen level was associated with changed iron homeostasis, the expressions of some iron metabolism-related key modulated genes, such as *Hepcidin*, *Fpn*, *DMT-1*, and *DCYTB*, were examined. It was found that the expressions of *Hepcidin* in liver and *Fpn*, *DMT-1* in duodenum significantly decreased at transcriptional level in OVX group than sham group. However, no difference existed in the expression of *DMT-1* (Fig. 2A and B).

### Effects of iron supplementation on serum E2, serum iron, tibial iron content, and iron particle in liver

**Fig. 3:**
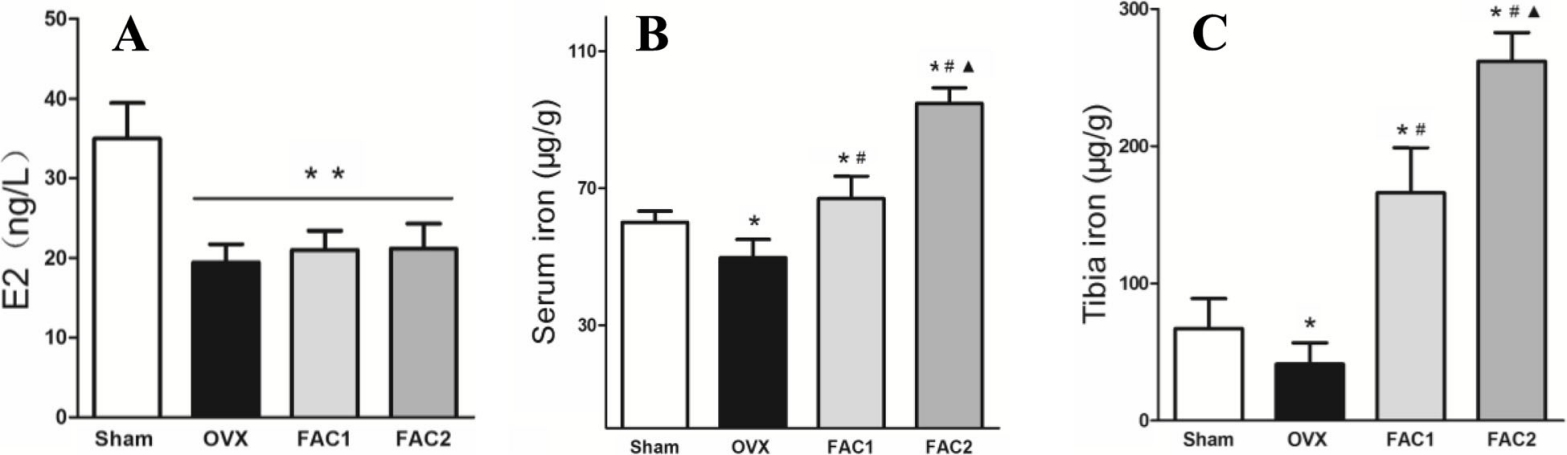
Parameters of serum E2 (**A**), serum iron (**B**), and tibial iron content (**C**) in sham and OVX rats. In OVX rats, 90 or 180 mg/kg ferric ammonium citrate (FAC) was administered as iron donor (FAC1 and FAC2). The bars represented mean ± SD. *p < 0.05 vs. sham, #*p* < 0.05 vs. OVX, ▲*p* < 0.05 vs. FAC1

**Fig. 4:**
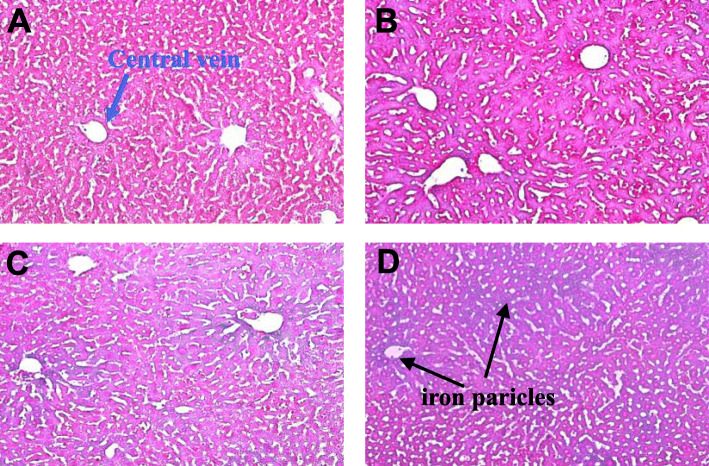
Histological detection of iron in liver by Perl’s staining. (**A**) Sham group; (**B**) OVX group; (**C**) FAC1 group; (**D**) FAC2 group. Original magnification ×100

As indicated in Fig. 3, FAC treatment seemed to have no effect on serum estrogen level. However, it indeed increased the serum and tibial iron levels in a dose-dependent manner, and iron staining in liver also complied with serum findings (Fig. 4).

### Micro-CT analysis of femur in each group

**Fig. 5:**
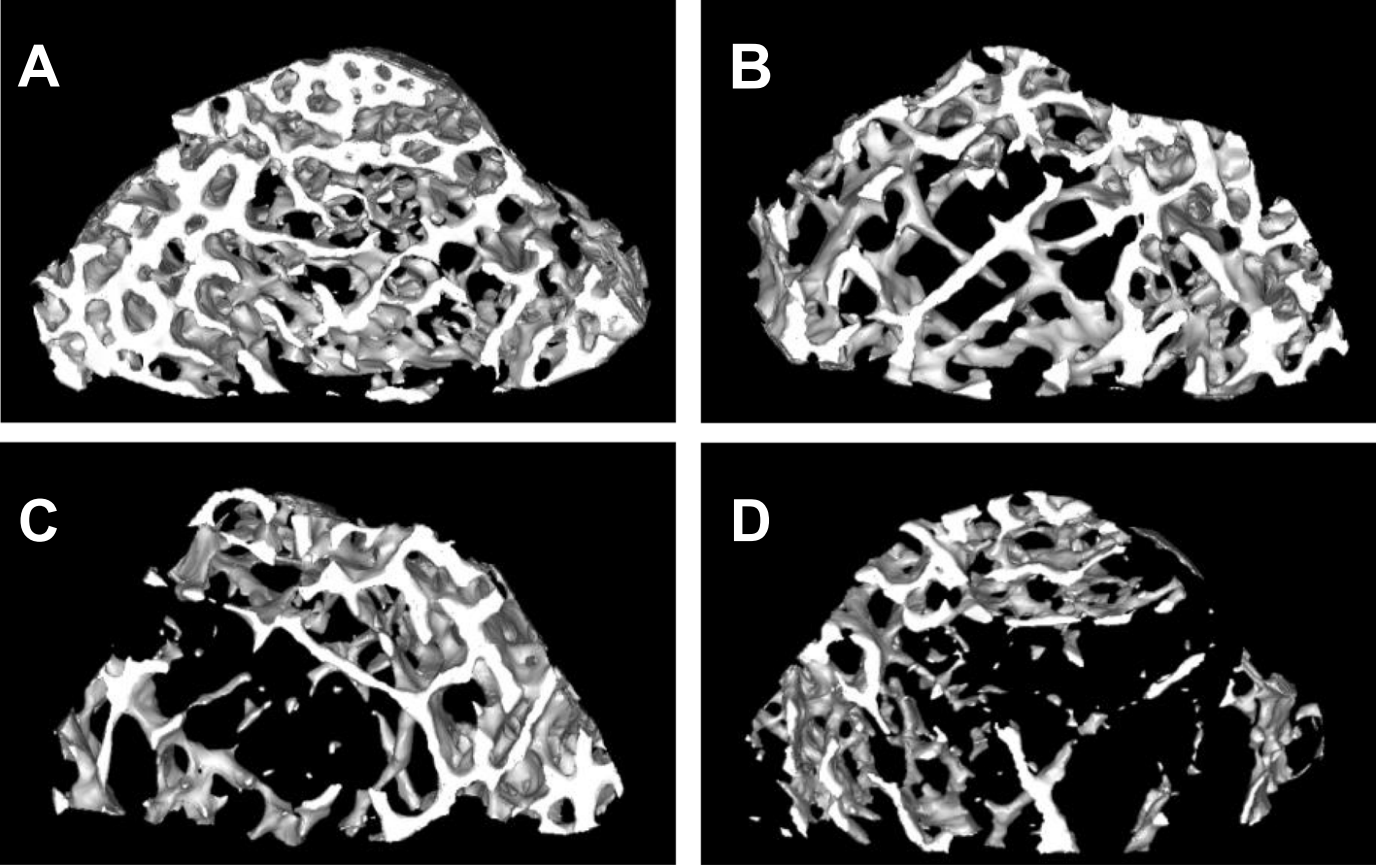
Three-dimensional micro-CT images of femur. (**A**) Sham group; (**B**) OVX group; (**C**) FAC1 group; (**D**) FAC2 group

MicroCT offers unique possibility to visualize microchanges in 3D sections. Trabecular bone microarchitecture was analyzed after reconstruction. As indicated in Table 2, the indices of BMD, BV/TV, Tb. th, Tb, and N in OVX group were lower than those in sham group, and these indices were further lower in FAC1 and FAC2 groups than OVX group (*p* < 0.05). Conversely, Tb, sp, SMI, and DA were significantly higher in OVX group than those in sham group. However, they were lower than those of FAC1 and FAC2 groups (*p* < 0.05). In addition, microCT supplied the information of 3D images of trabecular bone. Figure 5 provided convincing evidence that the number of trabecular bone decreased in iron-treated rats.

**Table 2:** Comparison of cancellous bone structural parameter

Groups	Sham	OVX	FAC1	FAC2
BMD (mg/mm)	420.3 ± 48.2	296.6 ± 51.9*	257.4 ± 64.8*^#^	204.8 ± 51.6*^#▲^
BV/TV (%)	57.35 ± 4.7	32.39 ± 5.2*	26.79 ± 6.0*^#^	20.47 ± 5.3*^#^
Tb.th (mm)	0.15 ± 0.08	0.13 ± 0.03*	0.11 ± 0.04*	0.10 ± 0.06*#
Tb.sp (mm)	0.15 ± 0.12	0.21 ± 0.08*	0.27 ± 0.14*	0.42 ± 0.10*^#▲^
Tb.N (N/mm)	3.81 ± 0.21	2.54 ± 0.18*	2.34 ± 0.11*^#^	1.95 ± 0.27*^#▲^
SMI	1.90 ± 0.13	2.15 ± 0.21*	2.45 ± 0.32*^#^	2.45 ± 0.16*^#^
DA	1.19 ± 0.41	1.32 ± 0.43	1.62 ± 0.24*^#^	1.43 ± 0.33*^#^

### Biomechanical testing

Values of mechanical properties of bone as maximum load, stress, and Young’s modulus were presented in Table 3. OVX resulted in a significant decrease in the maximum load, stress, and Young’s modulus as compared with the sham group (*P* < 0.05). These indices further decreased in FAC1 group versus OVX group, but there was no obvious significance. However, they sharply decreased in FAC2 group (*P* < 0.05). Meanwhile, significances of these three indices were also found between FAC1 and FAC2 group (*P* < 0.05).

**Table 3:** Mechanical parameters of diaphysial femur

Groups	Sham	OVX	FAC1	FAC2
Maximum load (*N*)	169.22 ± 14.35	163.39 ± 15.16*	158.16 ± 16.35*	150.92 ± 12.76*^#▲^
Maximum stress (MPa)	134.66 ± 16.42	130.02 ± 17.14*	125.86 ± 11.76*	120.10 ± 11.37*^#▲^
Young’s modulus (MPa)	6579.16 ± 645.38	4969.6 ± 452.13*	4426.6 ± 460.75*	3608.7 ± 442.63*^#^

Values were means ± SD. *p < 0.05 vs. sham, #*p* < 0.05 vs. OVX, ▲*p* < 0.05 vs. FAC1

### Effects of iron administration on serum BGP and β-CTX

**Fig. 6:**
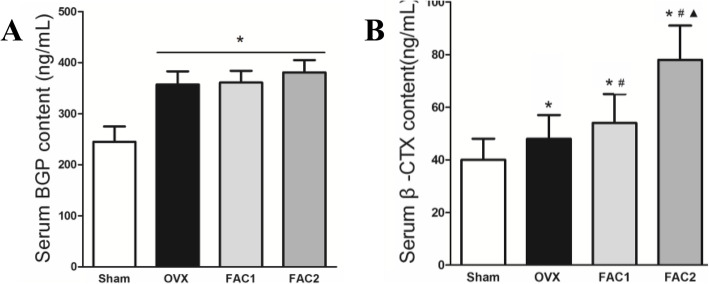
Plots of BGP and β-CTX in serum of various groups. Values were means ± SD. *p < 0.05 vs. sham, #*p* < 0.05 vs. OVX, ▲*p* < 0.05 vs. FAC1

The serum levels of bone turnover biomarkers, such as BGP and β-CTX, were examined. BGP, a bone formation marker, was significantly higher in OVX group than sham group. However, no difference was found with the dosing of iron (Fig. 6A). β-CTX was higher in OVX group. However, it was further higher in FAC1 and FAC2 groups (*P* < 0.05 for all comparisons) (Fig. 6B).

## Discussion

Osteoporosis could complicate iron overload related to hereditary or secondary hemochrotosis as reported in clinical [5, 17] and animal [18, 19] studies and suggested by in vitro studies as well [20–22]. In the present study, we examined the effect of ovariectomy on iron metabolism, treated ovariectomized rats with FAC, and examined the effect of iron on bone metabolism. Our study found that, not like female postmenopausal status, decreased estrogen after ovariectomy presented decrease of iron concentration in serum and tibial. After exogenous iron treatment, ovariectomized rat had a pronounced bone phenotype with abnormalities occurring in a dose-dependent fashion.

After ovariectomy, with declining estrogen, serum iron level, and tibial iron content decreased compared with sham group. This was consistent with another peer study reporting a close relationship between estradiol peak and small intestine iron absorption and transfer [23]. Some in vivo studies also confirmed that serum iron decreased after ovariectomy, and a subcutaneous dose of E2 at 40 μg kg^−1^ per day could compromise the declining of serum iron [13]. The mechanism may be that the changes of estrogen level could regulate the binding activity of iron regulatory proteins 1 (IRP1), at least in adipose tissue [14].

As to whether there was a direct connection between estrogen and iron metabolism, we detected the expression of iron-related genes in sham and OVX groups. The main sites of iron absorption are duodenum and upper jejunum. Inorganic dietary iron enters enterocytes via divalent metal transporter 1 (DMT-1) located in apical membrane of enterocytes. This transporter is selective for ferrous iron (Fe^2+^) from a reduction step of ferric iron (Fe^3+^, a predominant form in diet). In this process, duodenal cytochrome B (DCYTB) has been established as a major reductase [24]. Hepcidin, a peptide hormone produced in liver, is a key regulator of iron homeostasis. It acts by inhibiting cellular iron export through binding to ferroportin (FPN), a sole known cellular iron export channel, and subsequently inducing its degradation [25]. By this way, hepcidin inhibits iron absorption and iron transport for regulating plasma iron concentration and tissue iron distribution [26, 27]. The expression of hepcidin in liver decreased after ovariectomy. This result was consistent with the observation of Ikeda Y et al. [28] reporting that estrogen augmented the hepcidin expression through a GPR30-BMP-dependent mechanism.

Hence, our results confirmed that, not like female postmenopausal status, decreased estrogen induced by ovariectomy presented no elevation of iron concentration. Thus, for mimicking postmenopausal conditions, FAC was used as the donor of ferric ion as reported by Ishii et al. and Jia et al. [29, 30]. As expected, the administration of FAC to OVX rats could induce obvious iron accumulation, there were increased serum iron level and tibial iron content and stained iron particles were evident in liver (Table 2 and Fig. 1).

Based on these findings, we examined whether increased iron affected bone metabolism in OVX rats. There was a significant decrease of BMD in OVX rats and it further declined in iron treatment group. Furthermore, there was also a profound alteration of bone microarchitecture, and the decreases of BV/TV, Tb, th, Tb, and N and the increases of Tb, sp, SMI, and DA indicated a disorganization of trabecular microarchitecture [31]. In addition to micro-CT, mechanical testing was used to evaluate bone strength. Maximum load, maximum stress, and elastic modulus (Young’s modulus) are fundamental parameters for evaluating bone fragility [16]. In this study, 12 weeks of OVX resulted in reduced maximum load, maximum stress, and Young’s modulus. Consistent with the findings in micro-CT, these parameters further decreased in FAC1 and FAC2 groups, indicating a deterioration of bone strength.

In iron overload-induced osteoporosis models characterized by high levels of systemic iron and discrete trabecular at the distal ends of femurs, there was a significant increase in the serum levels of CTX, and it reflected the functionality of osteoclasts. However, no significant changes reflecting the activity of osteoblasts occurred in serum levels of BGP. Since normal skeletal homeostasis is dependent upon the coordination of osteoblast and osteoclast activities, several in vitro studies focused on the role of iron. Firstly, Yamasaki et al. found an iron dosed-dependent inhibition of proliferation and differentiation of osteoblasts [32]. Accordingly, Zarjou et al. suggested a role of ferritin and its ferroxidase activity in the inhibition of osteoblast activity [33]. It was also demonstrated that iron overload inhibited osteoblast activity in a concentration-dependent manner, and the underlying biological activity invoked by iron overload might be attributed to increased intracellular levels of reactive oxygen species (ROS) [34, 35]. Secondly, it is widely known that free iron could generate ROS or oxidative stress due to its ability of catalyzing Fenton reactions [36]. In addition, ROS may act as a mediator in RANKL-induced signaling pathways by facilitating the differentiation of osteoclasts [37]. Furthermore, E2 could induce antioxidant enzyme expression by stimulating the antioxidant defense system and inhibiting the formation of lipid peroxides in various tissues [38]. Thus, iron overload could further enhance increased ROS level induced by lowered estrogen in ovariectomized rats or postmenopausal women, accelerating net bone loss and leading to osteoporosis.

## Conclusion

In conclusion, not like female postmenopausal status, ovariectomized rats present no elevation of iron concentration. For mimicking the postmenopausal conditions of women to elucidate the effect of iron on bone metabolism, additional iron donors should be supplied, and iron overload further deteriorated osteoporosis mainly through accelerated bone resorption in ovariectomized rats. Thus, these data presented here may promote our understanding of the mechanism for postmenopausal osteoporosis and seek new ways of prevention and treatment.

## Data Availability

All the data pertaining to the present study are willing to share upon reasonable request.

## Abbreviations

OVX: Ovariectomy
FAC: Ferric ammonium citrate
BMD: Bone mineral density
PBS: Phosphate buffered saline
ICP-OES: Inductively coupled plasma-optical emission spectrometry
ROIs: Region of interest
IRP1: Iron regulatory proteins 1
DMT-1: Divalent metal transporter 1
DCYTB: Duodenal cytochrome B
FPN: Ferroportin
ROS: Reactive oxygen species
